# Prerequisites for primary care in obesity counselling and management: a quantitative, exploratory survey of general practitioners in the federal Republic of Germany

**DOI:** 10.1186/s12875-025-03048-w

**Published:** 2025-10-15

**Authors:** Julian Wangler, Michael Jansky

**Affiliations:** Centre for General Medicine and Geriatrics, University Medical Center of the Johannes Gutenberg, University Mainz, Am Pulverturm 13, Mainz, 55131 Germany

**Keywords:** Obesity, Overweight, General practitioner, Family doctor, Primary care, Treatment, Management

## Abstract

**Background:**

Obesity, overweight and their associated diseases pose serious challenges to the health system. General practitioners are in an especially favourable position to contribute to obesity prevention, make timely diagnoses, and initiate treatment in their patients. Beyond individual findings, the German-speaking world has a lack of studies giving a reliable reflection of the status quo for obesity management in primary care towards establishing common attitudes and behaviour patterns in treating this patient cohort. The aim of the present study was to determine the opinions, attitudes, experiences, and desires for improvement amongst GPs regarding obesity care. The aim of this study is to serve as a basis for developing an approach towards optimisation.

**Methods:**

All 13,912 GPs in active practice in Baden-Württemberg, Hesse, Rhineland-Palatinate, and Saarland between January and April 2024 were invited to take part in an online survey. A total of 4,038 fully completed questionnaires were included in the analysis; this corresponds to a response rate of 29%. Apart from descriptive analysis, Student’s t-test for independent samples was performed to determine significant differences between two groups.

**Results:**

The results have shown that most GPs see obesity as a major challenge that is clearly on the increase. Many of the GPs surveyed saw it as their responsibility to care for and provide therapeutic support for their overweight and obese patients. GPs especially prioritised assessing individual life situations surrounding their patients in the causes and consequences of overweight during weight counselling. Every second respondent provided dietary counselling, but fewer gave exercise counselling. Only some respondents used the opportunity to recommend specific health services to patients or refer their patients to these services. Respondents addressed dietary changes followed by psychosocial support and physical exercise issues. GPs were often unsatisfied with obesity management outcomes. This was worsened by general lack of time, resources, and connections to interprofessional support networks. Two groups in the sample stood out as drawing more optimistic conclusions after reflecting on their own counselling activities and therapeutic interventions. One group comprised GPs with specific additional qualifications, especially in diet and sports medicine. The other group consisted of GPs who regularly used digital health apps (DHAs) in their therapeutic approach; physicians in this group were markedly more satisfied with the results of disease management in their patients. Many respondents welcomed the introduction of the German *DMP Adipositas* obesity management programme and showed great interest in taking part in it. Many GPs expressed a desire for an easier overview of available local and regional health services.

**Conclusions:**

Even with the favourable conditions of primary care, the interview results indicate that the potential in primary care is currently not being fully exploited in overweight and obesity management. It would seem to make sense to raise awareness amongst GPs as to the circumstances of obesity while encouraging more motivational and behavioural consultation with patients. An obesity diagnosis should include actual recommendations on diet and exercise. Focussed diet and exercise counselling would also be welcome in primary care. GPs should also be encouraged to take on a role as mediators by referring patients to a broader healthcare network as necessary. Increased effort should be made towards developing structured, GP-compliant care programmes for obesity management towards implementing evidence-based treatment concepts adaptable to patient needs.

**Supplementary Information:**

The online version contains supplementary material available at 10.1186/s12875-025-03048-w.

## Background

The last few decades have seen a substantial increase in severe obesity issues in almost all Western countries [1–3]. This has gone so far that the WHO has begun referring to epidemic proportions in view of the sharp increase in obesity rates that are still one the rise [4]– [5]. A chronic disease limiting quality of life and presenting a high risk of morbidity and mortality, obesity may be the origin and amplifier for various other severe health conditions. Obesity is assumed to play a key role in around 80% of type 2 diabetes mellitus, around 35% of ischaemic heart disease, and around 55% of hypertensive disease cases in Europe alone [2, 6, 7]. Apart from that, overweight poses a burden on the musculoskeletal system while also promoting respiratory diseases, gallstones, and fatty liver disease [8, 9]. This clinical picture also causes considerable psychological stress for those affected [2, 3, 10–12]. Focusing on Germany, 53% of adults are overweight (BMI 25–29.9 kg/m²), 17% of whom are obese (BMI >30 kg/m²) [2, 7].

By far the most evidence-based guidelines see a multidisciplinary approach within a functioning support network as the most promising way of tackling the issue [13]. A multifactorial disease management system has therefore been recommended consisting of increased physical activity, a systematic reduction in calorie intake by changes in diet, and, where necessary, support in lifestyle and behavioural changes [1, 2]. The recommendations include longer-term steps after successful weight reduction to keep patients on track with their new weight and change in lifestyle.

Primary care is considered especially suitable for all these measures [14, 15]. General practitioners play a central role as they are usually familiar with their patients having treated them for many years. The robust relationship of trust between doctor and patient plays an especially important role in addressing severe obesity problems when the opportunity arises, initiating motivation towards lifestyle changes, and insuring adherence to treatment by management [16–18]. The patient perspective emphasises this: Surveys in various Western countries have shown patients affected by obesity to view their general practitioners as the first point of contact for advice on nutritional issues and physical activity, and also see a mediating role in their GPs for referral to other services [19–21].

In line with the above, general practitioners are in a position to liaise with other care and health services depending on individual needs while also providing their own counselling, support, and treatment. This involves the possibility of referring patients for consultation with diet and exercise therapy specialists or external services for psychosocial stabilisation or behavioural therapy [22–24]. Drug and surgical treatment options may also be considered [25]. German general practitioners can refer to the interdisciplinary S3 obesity guideline for preventive medicine, diagnostics, and treatment. Digital tools are also available to provide therapeutic support and contribute to prevention. Certified digital health apps (DiGA apps) have been available to doctors in Germany on prescription since 2020 [27–34].

Despite the great perceived potential in primary care, repeated indications of current deficits limiting the effectiveness of obesity care have arisen. One aspect applies to the occasionally documented negative attitudes towards patients with obesity, such as the assumption of lack of willpower [8, 26, 35–39]. This has led to subliminal stereotyping being common among physicians, which can lead to increased reservations on the effectiveness of diet or exercise therapies as well as insensitivity or inconsistency in medical communication [16, 21, 40–42]. Another issue difficulty may arise when general practitioners see their role only in referring patients to specialists and are unwilling to take on responsibility in helping the patient lose weight [24, 39, 43–45].

A lack of adequate support networks and programmes has also been given as a reason for the reticence occasionally raised for general practitioners in obesity management and a lack of funding for diet, exercise, and drug therapies from health insurance companies [38, 46, 47]. This has only just recently culminated in the dedicated *DMP Adipositas* obesity management programme [48]. To make matters worse, surveys in the USA, the UK and Germany have reported widespread challenges towards motivating obese patients to achieve sustainable weight change, which can lead to frustration on the part of physicians considering the scarcity of time and resources available in general medical care [17, 38, 49, 50]. It should be noted that there are hardly any studies on the possible effects of intervention by general practitioners such as in therapeutic treatment, so firm conclusions are difficult to draw on the general effectiveness of support from general practitioners in this area.

### Aim

Beyond individual international findings, the German-speaking world has a lack of studies giving a reliable reflection of the status quo for obesity management in primary care towards establishing common attitudes and behaviour patterns in treating this patient cohort. The aim of the present contribution was therefore to determine the opinions, attitudes, experiences, and desires for improvement amongst general practitioners regarding obesity care.

## Methods

This exploratory study collected detailed opinions and experiential accounts from general practitioners with a view to addressing the subject of this research project. A full survey of general practitioners in four federal states was conducted in the first half of 2024. This involved designing an online survey with a written cover letter sent in the regular mail.

### Survey method and development of questionnaire

A qualitative preliminary study surveying thirty-six general practitioners on the topic in 2022 [49] largely served as a basis in designing the survey used in this quantitative cross-sectional study (see Appendix 1). The development process also involved a detailed literature search on the topic [including 1, 2, 7, 8, 10, 14, 15–24, 26, 36, 37, 50].

The final questionnaire contained 43 questions and mainly comprised the following content areas:

- Perception of obesity as a healthcare problem and its causes.
- Role of general practitioners in care and treatment for patients with severe obesity issues, including willingness and suitability to take on responsibility.
- Experience with primary care and management in obesity care, including the reason or initiation for weight counselling, subject matter and focus in weight counselling; treatments, intervals, and regularity of weight counselling; weight loss targets; collaboration with other healthcare services.
- Challenges experienced and response strategies applied in obesity management and treatment.
- Satisfaction and outcome assessment regarding previous obesity management.
- Experience specifically with DiGA apps and obesity management guidelines.
- Favoured approaches and measures towards optimising obesity management in primary care.

Ordinal scales were widely used to achieve a good compromise between data quality and intuitive answerability in the survey for general practitioners as a target group with limited time to spare; many of these scales had four ratings to choose from. By working with this form of Likert scale, the aim was on the one hand to enable quick answering of the questions and on the other hand to avoid non-responses. Apart from the standardised questions, the survey included several open questions to reflect the exploratory nature of the study (questions 4, 7, 9, 14, 15, 19, 21, 25, 26, 28, 30, 38, 40, 42).

The sociodemographic characteristics recorded were gender, age, practice setting, type of practice, and patients per quarter. We performed a pretest before field use; this involved presenting the questionnaire to fifty randomly selected general practitioners among general practice lecturers in the general medicine faculty. The pretest showed answer categories to be easy to understand, well structured, and complete.

### Recruitment and sampling

All 13,912 general practitioners practicing in Baden-Württemberg (6,664), Hesse (3,839), Rhineland-Palatinate (2,667), and Saarland (742) were sent written postal invitations to take part in an anonymized survey; the invitation period lasted between January and April 2024. These federal states were selected as we aimed at including densely populated territorial federal states for at least an approximation to the primary care situation in Germany. The second reason was that the authors had current, complete contact lists due to continuous research into primary care in these federal states. This was a one-off mailshot where potential respondents were informed of password-protected access to the online survey (no incentives), inter alia.

A total of 4,038 completed questionnaires of the 4,114 questionnaires processed were included in evaluation (29% response rate). Table 1 compares the sample obtained against the reference data from the German Association of Statutory Health Insurance Physicians (KV) on the structure of general practitioners in Germany (see Table 1).

**Table 1:** Sample comparison to reference statistics

	Sample (*N* = 4,038)	Reference statistics
Gender:	63% male, 37% female	58% male, 42% female1
Average age:	53 (median: 53)	56 (median: 57)¹
Practice setting:	49% medium and large cities, 51% rural/small town	41% medium and large cities, 59% rural/small town¹
Type of practice:	54% individual practices, 35% group practices, 11% polyclinics or other establishments,	56% individual practices, 38% group practices, 6% polyclinics or other establishments²
Patients per quarter:	25% 500–1,500, 34% 1,501–2,000, 41% >2,000	Complete data unavailable
Physicians with relevant additional qualifications	681 (including diabetology: 23%, nutritional medicine: 27%, physical therapy: 7%, psychotherapy: 7%, social medicine: 9%, sports medicine: 30%)	Complete data unavailable

¹Based on health insurance research data in Rhineland-Palatinate (valid as of: 31 December 2021), available at: https://www.kv-rlp.de/institution/engagement/versorgungsforschung/

²Based on health insurance research data for Germany (valid as of: 31 December 2021), available at: https://gesundheitsdaten.kbv.de/

### Data analysis

We used SPSS 23.0 for data analysis. Student’s t-test for independent samples was used to determine significant differences between two groups. Two levels of significance were tested – mean difference at *p* < 0.05 and *p* < 0.001. This parametric method is considered statistically robust. We satisfied the necessary conditions with the number of cases, normal distribution in groups for differentiation, and samples originating from the same population [51].

We evaluated open questions using post-coding for qualitative content analysis. This involved creating a basal category system for answers in free text to each open question [52]. STROBE was used as the reporting statement.

## Results

### Perception of obesity as a healthcare issue and the role of primary care

According to the general practitioners surveyed, 19% of the patients currently in their care were severely overweight or obese (physicians in large and medium-sized cities: 17%; physicians in small towns and rural communities: 21%); of the physicians, 69% stated that this number had increased strongly or very strongly in the last five to ten years, 17% reported a moderate increase. Accordingly, 73% considered severe obesity problems to be a serious or very serious challenge to the healthcare system, while 24% saw it as a moderate or minor challenge.

The respondents saw the most common causes in inappropriate lifestyle due to family or social background, such as work and everyday stress, accompanied by unhealthy diet and lack of exercise (83%). 58% mentioned health problems resulting from physical predisposition or accompanying health problems such as chronic disease, whereas 45% gave personality predispositions as the main cause. Psychosocial issues played a central role in obesity development or perpetuation according to the opinion or experience of 64% of respondents.

65% of the physicians saw it as the main responsibility of general practitioners to act as the first point of contact for obesity management, treatment, and prevention compared to 27% who saw this is the main role for specialists supported by others such as general practitioners. The reasons that most of the respondents gave for the high suitability for general practitioners in dedicated care for obesity were their long-standing close familiarity with patients and the importance of communication in medicine in a holistic treatment approach.

33% of respondents stated that some or even most of their practice staff members had training in topics such as obesity, weight reduction, nutrition, exercise (17%, one staff member) for busy physicians to delegate tasks and improve patient care. Exactly half at 50% did not have any access to trained staff. Strikingly, all the respondents with additional qualifications in nutritional and/or sports medicine also mostly had staff members with additional specialist training.

Consultations with patients on overweight were usually brought up rather casually as part of another consultation and healthcare issues (82% frequently or occasionally); patients presented specifically for consultation on obesity far less frequently (27% frequently or occasionally).

### Experience with weight counselling

35% of respondents provided lifestyle and weight counselling frequently based on the total number of patients affected by obesity that each physician saw in daily practice; a further 48% provided this counselling occasionally and 17% rarely. Strikingly, physicians in urban environments conducted consultations more frequently than their colleagues in rural areas (91% vs. 74% frequently or occasionally, *p* < 0.001). Physicians with additional qualifications in fields such as diabetology, nutritional medicine, and sports medicine also provided weight counselling far more frequently.

54% of respondents stated that they mostly gave consultations at several points in time or continuously with patients returning for repeat appointments; 46% mostly gave one-off consultations. In this case, physicians with one or more of the additional qualifications surveyed performed regular consultations more often than average.

As this shows, general practitioners place special emphasis on assessing the life situation of patients for inclusion in the causes and consequences of obesity in weight counselling (see Table 2). Importantly, this involved briefing the patients on their options for weight reduction, making recommendations to suit the patient and, if necessary, agreeing on weight loss targets in a diet plan. Around every second respondent provided dedicated dietary counselling to patients or delegated this to specialists. The respondents gave specific exercise counselling less frequently. Respondents with relevant additional qualifications according to the corresponding aggregated query saw considerably greater importance in developing an individual weight loss strategy including follow-up as well as separate nutritional and exercise counselling compared to the greater respondent population. A diagnosis (according to guidelines) was also considered to be an important prerequisite for planning obesity management for patients affected. Some major differences also become apparent after dividing the sample into urban and rural physicians. Urban general practitioners placed greater emphasis on developing or agreeing on weight reduction strategies, whereas their colleagues in rural areas were especially concerned about addressing lifestyle issues in their patients, providing for psychosocial stabilisation and taking initial steps towards helping the patients to help themselves.

*“We don’t have many opportunities to delegate patients here in the countryside. That’s why we think it’s especially important for them to realise for themselves. They have to have the right mental and emotional mindset for that.”* Open answer from a responding general practitioner (female).

*“Who wants to be fat*,* who feels good about it? And then there are denial and compensation strategies*,* job stress*,* life’s hard knocks… […] It’s important to set the scenes first for people affected to develop the mental strength and inner willingness to accept fundamental change. In unacceptable situations*,* thirsty or hungry spells – literally. I’d call that empowerment.”* Open answer from a responding general practitioner (male).

**Table 2:** Areas of focus in weight counselling

Question: *Which of the following points do you think are especially important in weight counselling?* (*n* = 4,038, multiple answers possible)	Overall agreement	Urban vs. rural physicians	Additional qualifications queried, aggregated (*n* = 681)
Discussing the patient’s personal life situation	93%	85%/100%	99%
Clearly addressing the consequences of severe obesity	83%	95%/71%*	95%
General briefing on the principles of weight loss (gradual vs. acute weight loss)	82%	91%/73%*	94%
Possible causes of (personal) overweight	82%	74%/90%*	96%
Consultation on individual weight loss targets with follow-up	59%	70%/48%*	79%
Specific suggestions and recommendations to help the patient take the initiative	55%	65%/45%*	78%
Specific nutritional consultation (including switching to a low-calorie diet, eating habits and routines)	55%	65%/45%*	75%
Referral for further specialist care/treatment (specialist, nutritionist or, if necessary, specialised centre)	46%	53%/39%*	40%
(Guideline-compliant) diagnosis	45%	58%/32%*	73%
Psychosocial situation of the patient, stabilisation and improvement	45%	39%/52%*	57%
Dissemination of information materials with information sources	44%	37%/51%*	60%
Specific exercise consultation	37%	39%/34%	63%

Significant difference: *p < 0.001

54% of respondents stated that they had frequently or occasionally recommended specific offers to patients such as dietary counselling, health insurance services, sports courses, and local commercial weight reduction programmes such as Weight Watchers based on the total number of patients affected by obesity receiving lifestyle and weight counselling. Over a third at 36% stated that they had frequently or occasionally referred patients directly to specific healthcare services stating reasons such as collaborations that they had entered with these providers and services. The last point was raised considerably more often by physicians with qualifications in nutritional or sports medicine and mainly involved sports clubs, community exercise programmes, physiotherapists, and self-help groups (62%).

An open question (15) showed that patients often reacted with irritation, incomprehension, rejection, denial, or even open aggression when asked about their excess weight in the perception of many respondents. Respondents also often reported that patients reacted with ignorance or general apathy, which the physicians associated with psychosocial factors. The experiences of physicians were also negative after immediately suggesting weight loss or lifestyle changes. Around half mentioned low initial motivation and willingness.

### Experiences with (therapeutic) obesity management

Almost three-quarters of respondents (73%) stated that they frequently (27%) or occasionally (46%) took on responsibility for obesity management (11% rarely) and were thus involved in therapeutic measures in the medium to long term, reflecting the perceived role of obesity treatment and management in primary care. 46% of respondents claimed general or primarily responsibility for obesity management; a similarly large group at 43% followed instructions from specialists.

Results reflected responses to the question on general subject matter covered in weight counselling towards setting obesity treatment priorities (see Table 3). Respondents focused most heavily on diet change followed by psychosocial support and physical activation as specific management activities in their patients. Only a minority included adjuvant drug therapy as an important element in obesity treatment measures. Another question (27) showed similar results regarding surgical options, which only 20% of respondents considered appropriate. Once again, rural doctors placed more of an emphasis on psychosocial and educational elements, whereas their urban colleagues placed a heavier focus on active measures towards dietary and behavioural changes. Physicians with additional qualifications stood out clearly in the indicators determined.

**Table 3:** Areas of focus in obesity treatment

Question: *Where do you place your focus in planning therapy towards helping obese patients lose weight? Please rate the importance of the following elements in your practice.* (*n* = 4,038)	Overall agreement (Very/somewhat high taken together)	Urban vs. rural physicians (Very/somewhat high taken together)	Additional qualifications queried, aggregated (*n* = 681) (Very/somewhat high taken together)
Change in diet, diet therapy	58%	67%/49%*	80%
Psychosocial support	47%	40%/54%*	60%
Exercise therapy, promoting physical exercise, sports	43%	51%/35%*	69%
Organising information and materials for self-help	45%	39%/51%*	63%
Behavioural therapy (improving behavioural patterns and changing them in the long term)	40%	45%/35%*	62%
Adjuvant drug therapy (such as orlistat, liraglutide)	25%	27%/23%	28%

Significant difference: *p < 0.001

Well over a third of practitioners at 38% usually worked with several intermediate targets when drawing up a treatment plan while 45% typically defined a single intermediate target. In contrast, 85% of the respondents with additional qualifications used an approach with several intermediate targets.

### Challenges experienced and response strategies applied

The respondents were asked to evaluate medium and long-term effects of therapeutic management based on their previous experience. Only a minority at 40% were satisfied with the results of the therapeutic measures and reported very favourable (21%) or somewhat favourable (19%) outcomes. Physicians stating a primary focus on psychosocial support and promoting physical activity were proportionately more satisfied than the others. Likewise, physicians with most of their staff members with additional specialist training were more satisfied than those with few or no staff members with this training.

Most of the respondents at 61% had found it very difficult (32%) or somewhat difficult (29%) to motivate patients with obesity towards therapy, compared to just shy of a third at 32% finding it somewhat easy or very easy. Many general practitioners found it even more difficult to keep patients motivated during disease management towards achieving lifestyle changes or noticeable weight loss (35% very difficult, 30% somewhat difficult, 30% somewhat or very easy). Respondents also generally saw counselling and therapy adherence amongst patients with obesity as low (35% very low, 28% somewhat low, 34% somewhat high or very high). We were able to determine causal relationships that played a role in these challenges in handling this patient cohort and implementing structured and effective management strategies using various open questions (19, 21, 25):

- Lack of time resources, compatibility with daily practice (74%).
- Refusal to listen to advice, lack of motivation or compliance (65%).
- Problems maintaining a successful outcome (yo-yo effects, 56%).
- Lack of local opportunities and resources to initiate a multi-professional support network and collaboration with other healthcare services (53%, especially rural settings).
- Difficulties in setting and adjusting weight and lifestyle change targets appropriate for the patient (42%).

The open responses demonstrated that apart from lack of time and local resources, many general practitioners saw reduced willingness to seek advice and therapy amongst patients making effective intervention difficult. Many respondents reported that the lack of compliance and many setbacks they faced in obesity management that *“led to a feeling of frustration and exhaustion with these patients*.” Many respondents therefore showed a tendency towards viewing obesity not only as a clinical condition, but also as a complex problem resulting from acquired personality traits.

*“We see what a lack of self-efficacy feels like over and over in these patients.”* Open answer from a responding general practitioner (male).

*“The [patients in this] group fight too little and let themselves go too soon. That’s due to lifestyle*,* but psychological mechanisms also play a role.”* Open answer from a responding general practitioner (male).

*“I’m afraid there are many who are immune to any medical advice.”* Open answer from a responding general practitioner (male).

Remarkably, physicians with relevant additional qualifications experienced the challenges associated with obesity to a more moderate extent. Only 40% in this group found it very or somewhat difficult to persuade patients with obesity to undergo therapy. Similarly, only 41% of physicians with additional qualifications found it very or somewhat difficult to keep their patients motivated and on track in their disease management. One potential explanation for this could be that respondents with additional qualifications were considerably more likely to suggest procedures or strategies previously successfully used in motivating patients in the course of disease management (Question 26). Respondents with additional qualifications in psychotherapy, social medicine, and sports medicine in particular focused on stabilising their patients and boosting their self-esteem from the start. They also saw it as important to accommodate each patient’s own needs and expectations as far as possible when planning treatment.

*“That means consistently avoiding pressure to succeed and situations where they need to justify themselves. It’s more about meeting patients in their own reality and standards.”* Open answer from a responding general practitioner (male).

The priority is to provide a *“focused*,* concentrated jump-start”* (including a structured exercise and nutrition program) with individually appropriate motivational sports to “*set the scenes for consistent and steadfast weight loss*.” The respondents found it essential that the patient made the *“change from within”* and changed their habits. These respondents also saw health apps as beneficial for motivation and daily routine.

*“Long-term weight loss is achievable if you can get the patient to adopt this lifestyle change as a habit.”* Open answer from a responding general practitioner (female).

*“I see plenty of exercise and*,* most importantly*,* on a regular basis as the proper approach in combination with a healthy high-fibre diet. Patients need to be started on a corresponding programme; that will lead to success sooner or later as long as it is strictly adhered to.”* Open answer from a responding general practitioner (female).

Respondents also saw it as crucial to set realistic, precisely tailored targets in the therapy plan – targets that were neither too demanding nor too lax, beginning with gradual weight loss. This meant formulating clear and achievable final targets but also working with intermediate targets. Respondents also found arranging follow-up appointments and emphasising commitment to play an important role. Time intervals should ideally be kept at a few weeks, time to analyse reasons for failure in good time and to try out new approaches and similar.

*“How much time should I give myself to achieve which target*,* and in what way? If you don’t have that*,* you don’t have a compass to guide you in losing weight. […] That’s a quick way of consigning the whole thing to failure. […] You need criteria for success.”* Open answer from a responding general practitioner (male).

*“I take this approach: No hasty decisions. […] An individual programme for gradual and safe weight loss is much more important. […] And obviously there also needs to be certain targets for patients to pace themselves against.”* Open answer from a responding general practitioner (female).

Strikingly, many general practitioners with additional qualifications in nutrition and sports medicine reported close contacts with local fitness and healthcare promotion services. This applied to collaborations with gyms and fitness centres, self-help groups, and diet and health consultants. Respondents placed paramount importance in the ease of referring patients to trustworthy cooperation partners.

*“Many of us act as solo operators too much. You need to let that go. […] You need to see yourself within a network and a broader context. But that also means setting things up. […] It took us lots of time and energy to find these partners and sync up with them*,* but that clinched the deal for us.”* Open answer from a responding general practitioner (male).

These respondents ultimately saw sensitive communication and a collaborative approach to the doctor-patient relationship as important. They also saw it as essential to give these patients enough time for consultation while always remaining accessible to them, also in treatment setbacks.

*“Quite a few of these people suffer from depression in my experience. They often don’t believe they can overcome extreme weight problems. […] Become the architect of one’s own happiness*,* as it were. […] There are also ingrained routines that need to be addressed. […] That’s why doctors need to prepare for setbacks and think long-term. The most important thing is to take a patient*,* sympathetic approach without overtaxing the patient.”* Open answer from a responding general practitioner (female).

### Experiences with DiGA apps and guidelines

38% of respondents reported frequently (17%) or occasionally (21%) recommending digital health apps (DHAs or DiGA apps) to patients with obesity for support or prevention. This was far more common amongst urban doctors compared to their colleagues in rural settings (54% vs. 22%, *p* < 0.001). Under a third of physicians surveyed (29%) reported that they had already frequently or occasionally prescribed specialised DiGA apps to patients with obesity (61% were open to prescribing them in the future). Well over a third of urban physicians (42%) prescribed DiGA apps at least occasionally in contrast with only 13% amongst their rural colleagues in the sample (*p* < 0.001). Zanadio was the DiGA app most frequently prescribed amongst the dedicated obesity apps available in the DiGA directory.

Respondents with regular experience prescribing DiGA apps for obesity were considerably more optimistic about treatment results. In this group, 54% of respondents were (very) satisfied with the results of therapeutic measures compared to 34% from other respondents (*p* < 0.001). A considerably larger proportion of general practitioners with DiGA app experience rated counselling and therapy adherence in their patients with obesity as high or very high compared to other respondents (49% vs. 29%, *p* < 0.001), or found it easy or very easy to keep their patients motivated toward managing their obesity (48% vs. 27%, *p* < 0.001).

A detailed survey of potential favourable effects associated with the use of DiGA apps confirmed these findings (see Table 4). Apart from improved compliance, most respondents reported increased willingness to change behaviour as well as reduction in psychological problems due to app use; a considerable proportion of the weight loss achieved was attributed to DiGA app involvement.

**Table 4:** Beneficial effects observed from using DHAs (DiGA apps)

Question: *Which favourable effects on the health status have you already seen as a result of DiGA apps in your patients with obesity?* (*n* = 1,163, multiple answers possible)	Overall agreement
Increased compliance, such as taking their medications	83%
Improved health awareness and education	74%
Behavioural diet and lifestyle changes	72%
Increase mobility and joy of exercise	65%
Substantial weight reduction (such as BMI, abdominal circumference, waist circumference)	62%
Decrease in psychological issues and sequelae such as depression	60%
Improvement in self-management, such as in chronic disease	55%
Reduced complications such as hypoglycaemia	43%
Stable decrease in blood sugar (HbA1c)	19%
Prevention of complications such as diabetic foot syndrome and CHD	18%
Improvement in metabolic syndrome	17%
Avoidance of treatment escalation such as insulin therapy	11%

It became apparent over the course of the study that some general practitioners were not familiar with relevant (evidence-based) guidelines on obesity as a clinical condition. The best-known guidelines were the interdisciplinary S3 obesity prevention and therapy guidelines (56%) followed by the DEGAM practice recommendations for primary care in patients with obesity/overweight (42%). Half of the respondents used these recommendations frequently, and half used them occasionally.

### Optimising obesity management in primary care

We determined the approaches and measures favoured by general practitioners at the end of the survey towards improving treatment and management of patients with obesity in primary care and enhancing their effectiveness. Respondents increasingly called for the formation of more overarching support networks for obese patients on an outpatient or primary care basis. One area of focus lay on regional or local health networks and collaborations linking general practitioners, specialists, and other stakeholders in healthcare.

*“There has long since been an urgent need for greater official formalisation of the disease in view of the increasing number of overweight patients.”* Open answer from a responding general practitioner (male).

*“I see a lot of room for improvement*,* especially at local level*,* where there is too little collaboration*,* organisation*,* and supervision.”* Open answer from a responding general practitioner (female).

Many respondents also openly admitted a lack of any satisfactory overview of existing services or referral options in this area due to the lack of easy orientation. This was seen as another sensible reason for municipalities to prioritise bringing different services together and communicating these services to the public.

Respondents also generally criticised the considerable lack of support networks and healthcare services to provide continuous support to general practitioners in obesity prevention and management. Low-threshold counselling and motivational services were reported as especially lacking in rural areas, especially regarding psychosocial support. Respondents especially welcomed the recent decision to introduce an obesity disease management programme in Germany. Well over three-quarters of respondents (79%) considered this to make a very effective or fairly effective contribution to improving outpatient obesity care and management, including more standardisation, evidence-based care, improved cooperation between care levels, and patient education. Somewhat less than three-quarters of respondents at 71% rated their own interest in participating in the DMP Adipositas obesity programme as very high or somewhat high. Other suggestions included closer involvement from health insurance companies in (continuous) weight counselling for obese patients and a greater range of certified training courses for general practitioner practice staff, including the integration of specialised DiGA apps in obesity treatment and management.

Many respondents also expressed a desire for more efforts in primary prevention. Some general practitioners raised the criticism that demands for behavioural and situational prevention going back years had still not been implemented everywhere consistently, such as sugar tax as well as affordable balanced meals at daycare centres and schools. Respondents therefore saw it as the responsibility of primary care to prevent the development of severe overweight in their patients by alerting their patients to the risk factors early on and nurturing conditions for a healthy lifestyle. These respondents saw health checkups as an especially important ‘early warning system’ and had taken further training courses in nutritional medicine, amongst other specialities.

*“People now eating the wrong food and not exercising enough will be the obese patients of the future. That’s why the whole health system needs to take primary prevention far more seriously. We also need doctors strongly emphasising prevention and continually raising awareness among patients in their everyday lives. […] If nothing else*,* this’ll save us from lots of chronic illnesses and massive treatment costs.”* Open answer from a responding general practitioner (male).

This raises the question as to how patients only come to be the focus of healthcare after years of obesity. This raised some self-critical reflection in some of the physicians.

*“If someone’s obese*,* then something has already gone wrong on the physician’s part. These people should have been noticed earlier and taken care of accordingly. So anything that might help us identify these people earlier would be welcome.”* Open answer from a responding general practitioner (female).

This further emphasises the importance of regular patient contact as well as thorough and consistent patient education. Continuous bloodwork would also help identify early risk factors for general practitioners to watch out for. Some of the respondents expressed the opinion that practice staff involvement and training could be put to more use in supporting general practitioners.

*“We still definitely have some catching up to do in Germany towards providing special training for staff members and involving them as an asset in providing care for certain groups. This’ll take the burden off general practitioners and ensure greater effectiveness in primary care. Especially in obesity*,* I see a relatively serious need to catch up.”* Open answer from a responding general practitioner (female).

## Discussion

### Principal findings

Primary care settings are in a favourable position to provide obesity counselling and management. The survey results show that most general practitioners experienced obesity as a major and challenge that was clearly on the increase. This applied both to the healthcare system and everyday activities in general practice offices. Many of the general practitioners saw it as their own responsibility to care for and, where necessary, provide therapeutic support for their overweight and obese patients. General practitioners especially prioritised assessing individual life situations affecting their patients in the causes and consequences of overweight during weight counselling. The following sets the varied range of priorities: Every second respondent provided dietary counselling, but fewer gave exercise counselling. Urban general practitioners focused more on agreeing weight reduction strategies; their rural colleagues were especially focused on patient lifestyle as well as stabilisation and self-help. Only some respondents used the opportunity to recommend specific health services to patients or refer their patients directly to these services.

Respondents addressed dietary changes followed by psychosocial support issues and encouraging physical exercise. General practitioners often drew mixed conclusions on obesity management. Many were finding treatment adherence and motivation in patients with obesity to be limited. This was worsened by general lack of time, resources, and connections to interprofessional structures, especially in rural settings.

Two groups in the sample stood out as drawing more optimistic conclusions with fewer challenges in performing effective obesity management after considering their own counselling activities and therapeutic interventions. One group comprised general practitioners with specific additional qualifications, especially in diet and sports medicine. These respondents named successful strategies in dealing with this patient cohort noticeably more often than other physicians; strategies included individual, needs-oriented and multi-stage weight loss strategies with regular follow-up appointments as well as separate nutrition and exercise counselling. Strikingly, many of these respondents also reported regular collaboration with other healthcare services and consistently referred their patients to these services. The other group consisted of general practitioners who regularly used DiGA apps in their therapeutic approach; these respondents were markedly more satisfied with the results of disease management in their patients. Generally, physicians with experience in prescribing digital health apps reported a striking number of favourable health effects in the treatment of obesity using DiGA apps.

Only some of the respondents were familiar with obesity-related guidelines; an even smaller percentage used them regularly. Many respondents welcomed the introduction of the German DMP Adipositas obesity management programme and showed great interest in taking part in it. This tallies with their call for more (evidence-based) support networks in this health area. The respondents also identified further approaches towards optimising outpatient and primary care-based obesity management ranging from closer involvement from health insurance companies to improved training programmes for GP practice staff to reinforce general (nutritional) preventive care. Many general practitioners expressed a desire for an easier overview of health services available locally and regionally that they could refer their patients to without much additional workload.

### Comparison with prior work

Overall, the results obtained match the existing study situation in that outpatient physicians face multiple hurdles and challenges in treating obesity, some of which are directly related to patients and some to external factors. Obesity is therefore a highly polarising clinical condition for some physicians; this gives rise to varying degrees of willingness to provide care and support depending on personal experience and basic attitude [11, 12, 14, 16, 25, 38, 44]. A previous qualitative preliminary study has already shown how varied these can be [49]. This study involved interviewing thirty-six general practitioners and categorising them into four clusters or archetypes. The first cluster was widely represented in the sample; physicians in this cluster were especially conspicuous through their negative attitude towards obesity management in primary care, patients affected, willingness to provide treatment, and assumptions on options for personal efficacy. The other types were open-minded and actively committed, albeit to varying degrees. One of these active groups focused more on early and consistent dietary adjustment and exercise, while another group concentrated more on collegial and collaborative psychosocial support. Especially worth emphasising are the respondents that were integrated into informal networks with local gyms and exercise services or psychosocial and behavioural therapists depending on their chosen approach. The present quantitative study also includes all these areas of focus supplemented by additional findings.

International studies have so far indicated that general practitioners are aware of the importance of overweight and obesity management [37]. Even so, their attitudes towards severely overweight patients are often affected by scepticism [8, 26, 36, 42–44, 49, 50, 53]. Indeed, some general practitioners show a corresponding lack of trust in the effectiveness of diet or exercise therapy. Subtle, or possibly even overt stereotyping renders obesity-related medical communication insensitive and inconsistent, which can be extremely disconcerting for patients [16, 54]. General practitioners often seem to prefer to take a more passive role, viewing weight reduction as mainly the responsibility of their patients [43, 44]. This leads to largely ad-hoc weight reduction programmes designed for each case as it stands [24, 42]. In a Hungarian study on the same topic clear relation was found between the own BMI of doctors and their habits and practice. Doctors in the normal BMI range were unanimous that they should be a model for their patients [55]. There is also some evidence that general practitioners are relatively unaware of obesity-based guidelines and therefore rarely use them. Several studies have therefore concluded that obesity diagnostics and management in primary care do not currently comply with common guideline recommendations [13, 56, 57]. This is supported by a cohort study of 209 patients with obesity that attended primary care consultations, which shows that only 25% of the clinical records met al.l the criteria established in the therapeutic guidelines regarding diet prescription [58].

The lack of adequate structures and programmes has also been repeatedly discussed as a reason for reticence amongst general practitioners in obesity management [38, 46, 50]. This has resulted in a lack of evidence-based treatment concepts that can also be tailored to fit the patient towards continuous support from their general practitioners in lifestyle changes [3, 12, 36, 40, 50]. Many general practitioners therefore feel left on their own in shouldering the all-encompassing and long-term responsibility that is obesity management. The primary care community generally sees DMP Obesity as one way to overcome this longstanding deficit [41, 46].

Awareness of local assistance and support services for patient referral is often limited with only a small proportion of general practitioners consistently making use of these services, such as by informing their patients of fitness and exercise services or psychotherapeutic intervention [17]. Many physicians seem to lack either awareness of the general picture or low-threshold referral options as clearly evidenced in the results from the survey in the present contribution [59].

Many of the issues addressed are reflected in the patient perspective; studies have shown that patients view their general practitioners as central and trustworthy points of contact for advice, support, and therapy [50]. Several studies have indeed found severely obese patients to show increased willingness to make lifestyle changes after weight counselling with their own general practitioners [20, 45]. In clinical practice, however, severely overweight patients are often dissatisfied with the care that their general practitioners provide [24, 40]. Studies have shown that obesity is mostly an incidental diagnosis, or that the subject comes up later than it should. Discussions about the weight situation are mostly not held regularly, and there is no continuous support. This leads to patients often feeling alone with their overweight problems [60]. Apart from that, only some general practitioners combine overweight and obesity diagnoses with specific advice or instructions on diet and physical activity, and only agree with the patient on specific targets or time frames in some cases [22, 24, 50]. Respondents articulated a desire for referrals to support services, whether in the form of health insurance services, gym classes, or self-help groups, but are only given this support relatively rarely. We identified several problem areas in primary care during an interview study on patients with obesity [53]: 1: Incidental or delayed diagnosis of obesity; 2: Lack of continuous weight counselling; 3: Lack of agreement on specific weight-loss targets; 4: Lack of referrals to assistance and support services; 5: Insensitive or stigmatising consultation. Another problem from the patient perspective is motivation. A recent survey of 209 primary care patients showed that a majority (68%) of the respondents reported not having sufficient motivation to adhere to a weight loss program [61]. The barriers to adherence to diet and exercise plans most frequently mentioned by patients were not having a prescribed diet, joint pain, getting tired or bored of dieting and laziness. Both the high percentage of patients reporting insufficient motivation to lose weight and the barriers to weight loss identified suggest that patients feel the need to improve their motivation, which should be promoted through primary care.

The ‘Awareness, Care and Treatment In Obesity MaNagement’ (ACTION) Study was the first US nationwide study to investigate barriers to obesity management from the perspective of people with obesity, health care professionals and employers [62]. The results showed that despite changing attitudes toward obesity and its increasing recognition as a chronic, serious, and progressive disease, many barriers to effective care remain, with the consequence that few people with obesity are seeking and receiving long-term obesity care. Therefore, the ACTION Study highlights a need for massive collaborative efforts that can lead to better understanding of obesity and effective solutions for obesity care. Further ACTION studies were conducted in other countries (including Canada, Southeast Asia, Switzerland) and came to similar findings [60, 63, 64].

Finally, it should be noted that various differences between rural and urban physicians were identified during the study. This was also the case in the qualitative preliminary study [49]. Several other studies have found that rural physicians prioritize patients differently due to different structural conditions [2, 7, 8, 10, 14–24, 27, 33, 49, 53]. Specialist networks and healthcare providers are often significantly less available or even non-existent in rural areas, so primary care physicians try to compensate for this by practicing more holistic approaches to care. This explains, for example, why primary care physicians, when caring for patients with obesity, focus more on the individual’s personal life situation and the causes of obesity. A variety of approaches emerge for optimising obesity management in primary care from the study results, taking the literature into account:

- Recent studies have emphasised the immense value and indispensable role of general practitioners in managing weight issues towards effectively counteracting the global spread of overweight and obesity [15].
- It would seem sensible to raise awareness amongst general practitioners that obesity is a disorder with a complex background involving not only individual lifestyle factors but also external factors such as life circumstances, genetic predisposition, and pre-existing conditions [1, 2, 7, 16].
- Obesity issues should be routinely, consistently, and promptly addressed in primary care. Checkups provide an ideal setting [36, 37, 41].
- Diagnosing severe overweight or obesity should be combined with specific action recommendations and realistic targets set for each patient in diet and exercise. Existing guidelines provide additional assistance and guidance [10, 65].
- Diet and exercise counselling would seem to make a sensible contribution towards reinforcing obesity prevention in primary care. Good international practice examples and compact models for implementation have already been proposed to this effect [2, 23, 65–69]. Staff at the doctor’s office could also be enrolled for support and given targeted training.
- Regular counselling sessions with commitments and a strategy of engaging with patients in their personal situation (behaviour-oriented treatment strategies) together with continuous patient motivation are important prerequisites for the long-term success of obesity management [21, 22, 24].
- General practitioners should also be encouraged to take on a role as mediators by referring patients with obesity to a broader healthcare network as necessary. Almost all statutory health funds in Germany provide prevention programmes; the same applies to health authorities, which often provide a useful guide to the local and regional training and consultation services available [8, 47]. Local health promotion networks could provide a high level of additional value in providing general practitioners with an overview of existing health services for referring patients to the services they need [70, 71].
- Establishing and developing structured care programmes for obesity management would seem sensible [70, 72, 73]. The reality of primary care also needs special consideration. These programmes should not only aim towards improving patient care but also training for general practitioners. This would allow the added benefit of digital tools such as DiGA apps to be passed on [27, 74, 75] while also paving the way for effective staff training. Strategies for evidence-based support for general practitioners may further improve how they provide care. Here, it would seem important to raise awareness of the value of reviewed and certified guidelines [56, 57, 76, 77]. International model projects for holistic practice staff training may provide guidance and could be adapted to suit the specific situation [7, 20, 35, 38, 41]. Focusing on long-term step-by-step programmes taking full account of patients as human beings in their respective sociocultural environments would be a productive way of addressing the complexities of overweight and obesity [73].
- Targeted training in nutrition and sports medicine may help provide a more accurate response to patients affected by obesity and start them on therapy more effectively [20, 22, 36].

Ideally, general practitioners would be placed in a position where they can fulfil two main tasks – individual consultation and treatment as well as coordination within a multidisciplinary obesity care network. This includes not only specific training for those giving treatment and improved but also more structured networking amongst various service providers; remuneration commensurate to the services provided is just as important.

### Strengths and weaknesses

The survey developed from a qualitative preliminary study and was therefore tailored to the perspective of primary care in a specific real-life approach. We were able to collect non-standardised responses from a series of open questions. The survey achieved a relatively high response rate, allowing us to diversify the sample in broad terms of characteristics and attitudes towards the study topic.

Even so, there are limitations. The study cannot claim to be representative in the strict sense due to the limited number of cases and regional recruitment strategy, at most approximating to a representative study due to comparison with health insurance data. It is also possible that physicians with an interest in the topic participated more closely in the study.

We would also like to note that these are the viewpoints, reported experiences, and opinions of the general practitioners surveyed on securing effective primary care. In order to capture these perspectives and experiences as openly and comprehensively as possible, and thus emphasize the general practitioner perspective, we deliberately avoided a narrow theoretical framework. Nor did we prescribe a specific or restricted definition of severe overweight or obesity, as our goal was to capture the general perspective in primary care. This approach, of course, also has clear disadvantages, as it results in a lack of clarity in our study’s examination of the clinical picture. Nevertheless, the focus was on the everyday assessments of general practitioners and terms such as ‘severe overweight’ and ‘obesity’ have deliberately not been clearly demarcated from each other.

This paper has been framed in the context of a classic narrative of obesity with regrad to primary care in Germany. From this perspective, individual nutrition and exercise are more central, whereas the combination of behavioural, psychological, pharmacotherapy and surgery as evidence-based therapies is given less focus.

The scaling method used in the questionnaire is another point of criticism. This was not always consistent, such as in the scale levels used or additional categories included such as “undecided” or “difficult to say.” This was due to the authors’ efforts to ensure that the survey was as easy to complete as possible given the time constraints applicable to the target group.

## Conclusions

General practitioners see obesity as a rapidly increasing condition and a challenge to healthcare; their experiences are ambivalent. Even so, many general practitioners see themselves as the first point of contact for this patient cohort. They place divergent emphases on obesity management, which may also vary according to additional qualifications as well as urban and rural settings. Physicians focused most heavily on changes in diet followed by psychosocial support issues and encouraging physical exercise in their patients. DMP Obesity was especially welcomed in view of the existing deficit in evidence-based, interprofessional care services, and the desire for further expansion of municipal support networks was also brought up.

One of the key findings in the current contribution was physicians with corresponding additional qualifications (especially nutritional and sports medicine) well as those with experience in DiGA-certified health apps reported obesity management issues to a far lesser extent; satisfaction with the results of the therapeutic intervention was more favourable. In the primary care setting, the systematic use of digital tools is conceivable to support patients in changing their lifestyles. The same applies to general networking with other services and the use of evidence-based instruments.

## Supplementary Information

Supplementary Material 1.

## Data Availability

All data generated or analysed during this study are included in this published article.

## Abbreviations

GP(s): General Practitioner(s)

## References

[1] Durrer Schutz D, Busetto L, Dicker D, et al. European practical and patient-centred guidelines for adult obesity management in primary care. Obes Facts. 2019;12(1):40–66. 10.1159/000496183. 30673677 10.1159/000496183 PMC6465693

[2] Klein S, Krupka S, Behrendt S. Weißbuch Adipositas – Versorgungssituation in Deutschland [Obesity white paper – care situation in Germany]. Berlin: MVG; 2016.

[3] WHO Europe. The challenge of obesity in the WHO European region and strategies for response. Kopenhagen: WHO; 2007.

[4] WHO. WHO European Regional Obesity Report. 2022. 2022. https://www.who.int/europe/publications/i/item/9789289057738 [Cited 2024 Okt 10].

[5] WHO. New WHO report: Europe can reverse its obesity. epidemic. 2022. https://www.who.int/europe/news/item/03-05-2022-new-who-report--europe-can-reverse-its-obesity--epidemic [Cited 2024 Okt 10].

[6] Prospective Studies Collaboration. Body-Mass-Index and cause specific mortality in 900000 adults: collaborative analysis of 57 prospective studies. Lancet. 2009;373(9669):1083–96. 10.1016/S01406736(09)60318-4. 19299006 10.1016/S0140-6736(09)60318-4 PMC2662372

[7] Mensink GB, Lampert T, Bergmann E. Overweight and obesity in Germany 1984–2003. Bundesgesundheitsblatt - Gesundheitsforschung – Gesundheitsschutz. 2005;48(12):1348–56. 10.1007/s00103-005-1163-x. 16270188 10.1007/s00103-005-1163-x

[8] Huang J, Yu H, Marin E, Brock S, Carden D, Davis T. Physicians‘ weight loss counseling in two public hospital primary care clinics. Acad Med. 2004;79(2):156–61. 10.1097/00001888-200402000-00012. 14744717 10.1097/00001888-200402000-00012

[9] The GBD 2015 Obesity Collaborators. Health effects of overweight and obesity in 195 countries over 25 years. N Engl J Med. 2017;377(1):13–27. 10.1056/NEJMoa1614362. 28604169 10.1056/NEJMoa1614362 PMC5477817

[10] Anderson DA, Wadden TA. Treating the obese patient. Suggestions for primary care practice. Arch Fam Med. 1999;8(2):156–67. 10.1001/archfami.8.2.156. 10101987 10.1001/archfami.8.2.156

[11] Puhl RM, Heuer CA. The stigma of obesity: a review and update. Obesity. 2009;17(5):1–24. 10.1038/oby.2008.636. 10.1038/oby.2008.636 19165161

[12] Ashmore JA, Friedman KE, Reichmann SK, Musante GJ. Weight-based stigmatization, psychological distress, & binge eating behavior among obese treatment-seeking adults. Eat Behav. 2008;9(2):203–9. 10.1016/j.eatbeh.2007.09.006. 18329599 10.1016/j.eatbeh.2007.09.006

[13] Semlitsch T, Stigler F, Jeitler K, et al. Management of overweight and obesity in primary care-A systematic overview of international evidence-based guidelines. Obes Rev. 2019;20(9):1218–30. 10.1111/obr.12889. 31286668 10.1111/obr.12889 PMC6852048

[14] Whitlock EP, Orleans CT, Pender N, Allan J. Evaluating primary care behavioral counseling interventions. Am J Prev Med. 2002;22(4):267–84. 10.1016/s0749-3797(02)00415-4. 11988383 10.1016/s0749-3797(02)00415-4

[15] Jansen S, Desbrow B, Ball L. Obesity management by general practitioners: the unavoidable necessity. Aust J Prim Health. 2015;21(4):366–8. 10.1071/PY15018. 26349423 10.1071/PY15018

[16] Brown I, Thompson J, Tod A, Jones G. Primary care support for tackling obesity: a qualitative study of the perceptions of obese patients. Br J Gen Pract. 2006;56(530):666–72. 16953998 PMC1876632

[17] Tham M, Young D. The role of the general practitioner in weight management in primary care – a cross sectional study in general practice. BMC Fam Pract. 2008;9:66. 10.1186/1471-2296-9-66. 19077319 10.1186/1471-2296-9-66 PMC2614998

[18] van Dijk L, Otters HB, Schuit AJ. Moderately overweight and obese patients in general practice: a population based survey. BMC Fam Pract. 2006;7:43. 10.1186/1471-2296-7-43. 16827937 10.1186/1471-2296-7-43 PMC1564048

[19] Gstettner A, Holzapfel C, Stoll J, Hauner H, Gewichtsreduktion. Evaluation der Möglichkeiten auf hausärztlicher versorgungsebene und der patientenzufriedenheit [Weight reduction: evaluation of the possibilities in primary care and patient satisfaction. Results from a weight reduction trial]. Dtsch Med Wochenschr. 2013;138(19):989–94. 10.1055/s-0033-1343164. 23633277 10.1055/s-0033-1343164

[20] Loureiro ML, Nayga RM, Rodolfo Jr. Obesity, weight loss and physician‘s advice. Soc Sci Med. 2006;62(10):2458–68. 10.1016/j.socscimed.2005.11.011. 16376006 10.1016/j.socscimed.2005.11.011

[21] Sturgiss E, Haesler EM, Elmitt N, van Weel C, Douglas K. Increasing general practitioners’ confidence and self-efficacy in managing obesity: a mixed methods study. BMJ Open. 2017;7(1):e014314. 10.1136/bmjopen-2016-014314. 28132016 10.1136/bmjopen-2016-014314 PMC5278274

[22] Wiesemann A, Barlet J, Engeser P, Kuth N, Müller-Bühl U. Verhaltensorientierte beratungsstrategien für die hausarztpraxis [Obesity behavior change promotionin primary Care]. Z Allg Med. 2006;82(3):103–7. 10.1055/s-2006-921505.

[23] Lau D, Klein R, Steinkohl M. Prävention durch Fokussierte Ernährungsberatung in der hausarztpraxis [Prevention through focused nutritional advice in general practice]. Z Allg Med. 2003;79(5):234–7. 10.1055/s-2003-40714.

[24] Simkin-Silverman LR, Gleason KA, King WC, et al. Predictors of weight control advice in primary care practices: patient health and psychosocial characteristics. Prev Med. 2005;40(1):71–82. 10.1016/j.ypmed.2004.05.012. 15530583 10.1016/j.ypmed.2004.05.012

[25] Roebroek YGM, Talib A, Muris JWM, et al. Hurdles to take for adequate treatment of morbidly obese children and adolescents: attitudes of general practitioners towards conservative and surgical treatment of paediatric morbid obesity. World J Surg. 2019;43(4):1173–81. 10.1007/s00268-018-4874-5. 30478687 10.1007/s00268-018-4874-5

[26] Jay M, Kalet A, Ark T, et al. Physicians’ attitudes about obesity and their associations with competency and specialty: a cross-sectional study. BMC Health Serv Res. 2009;9:106. 10.1186/1472-6963-9-106. 19552823 10.1186/1472-6963-9-106 PMC2705355

[27] Wangler J, Jansky M. Welche potenziale und mehrwerte Bieten DiGA für die Primärversorgung? - Ergebnisse einer befragung von Hausärzt*innen in Deutschland [What potential and added value do DiGA offer for primary care?-Results of a survey of general practitioners in Germany]. Bundesgesundheitsblatt - Gesundheitsforschung – Gesundheitsschutz, 65 (12): 1334–43. 10.1007/s00103-022-03608-w 10.1007/s00103-022-03608-w PMC9722862 36269336

[28] Ernsting C, Stuhmann LM, Dombrowski SU, et al. Associations of health app use and perceived effectiveness in people with cardiovascular diseases and diabetes: population-based survey. JMIR Mhealth Uhealth. 2019;7(3):e12179. 10.2196/12179. 30920383 10.2196/12179 PMC6458532

[29] Brandt CJ, Søgaard GI, Clemensen J, Sndergaard J, Nielsen JB. General practitioners’ perspective on ehealth and lifestyle change: qualitative interview study. JMIR Mhealth Uhealth. 2018;6(4):e88. 10.2196/mhealth.8988. 29666045 10.2196/mhealth.8988 PMC5930171

[30] Stern AD, Brönneke J, Debatin JF, et al. Advancing digital health applications: priorities for innovation in real-world evidence generation. Lancet Digit Health. 2022;4(3):e200–6. 10.1016/S2589-7500(21)00292-2. 35216754 10.1016/S2589-7500(21)00292-2

[31] König IR, Mittermaier M, Sina C, et al. Evidence of positive care effects by digital health apps—methodological challenges and approaches. Inn Med. 2022;63(12):1298–306. 10.1007/s00108-022-01429-2. 10.1007/s00108-022-01429-2 36279007

[32] Kuhn E, Rogge A, Schreyer K, et al. Apps on prescription in the medical office, but how? A case-based problem outline of medical-ethical implications of DHA usage. Gesundheitswesen. 2022;84(8/9):696–700. 10.1055/a-1473-5655. 33957698 10.1055/a-1473-5655

[33] Wangler J, Jansky M. Gesundheits-Apps Als instrumente der Prävention? – Eine interviewstudie Zu potenzialen für Das hausärztliche setting [Health apps as instruments of prevention? – a qualitative study on the potential for the primary care setting]. Präv Gesundheitsf. 2020;15(4):340–6. 10.1007/s11553-020-00769-x.

[34] Goeldner M, Gehder S. Digital Health Applications (DiGAs) on a Fast Track: Insights From a Data-Driven Analysis of Prescribable Digital Therapeutics in Germany From 2020 to Mid-2024. J Med Internet Res. 2024;26:e59013. 10.2196/59013. 39208415 10.2196/59013 PMC11393499

[35] Wiesner S. Leitliniengerechte Therapie der Adipositas im Kontext der Versorgungsmöglichkeiten [Guideline-based treatment of obesity in the context of care options]. In Adipositas und Public Health. Rahmenbedingungen, interdisziplinäre Zugänge und Perspektiven für erfolgreiche Präventionsstrategien [Obesity and Public Health. Framework conditions, interdisciplinary approaches and perspectives for successful prevention strategies]. Edited by Heintze C. München: Juventa; 2010: 41–64.

[36] Brotons C, Ciurana R, Pineiro R, et al. Dietary advice in clinical practice: the views of general practitioners in Europe. Am J Clin Nutr. 2003;77(4 suppl):1048S-1051S. 10.1093/ajcn/77.4.1048S. 12663317 10.1093/ajcn/77.4.1048S

[37] Bocquier A, Verger P, Basdevant A, et al. Overweight and obesity: knowledge, attitudes, and practices of general practitioners in France. Obes Res. 2005;13(4):787–95. 10.1038/oby.2005.89. 15897489 10.1038/oby.2005.89

[38] Metz U, Welke J, Esch T, et al. Perception of stress and quality of life in overweight and obese people – implications for preventive consultancies in primary care. Med Sci Monit. 2009;15(1):1–6. 19114978

[39] Fogelman Y, Vinker S, Lachter J, et al. Managing obesity. A survey of attitudes and practices among Israeli primary care physicians. Int J Obes Relat Metab Disord. 2002;26(10):1393–7. 10.1038/sj.ijo.0802063. 12355337 10.1038/sj.ijo.0802063

[40] Ruelaz A, Diefenbach P, Simon B, et al. Perceived barriers to weight management in primary care- perspectives of patients and providers. J Intern Med. 2007;22(4):518–22. 10.1007/s11606-007-0125-4. 10.1007/s11606-007-0125-4 PMC1829430 17372803

[41] Sturgiss E, Jay M, Dampbell-Scherer D, et al. Challenging assumptions in obesity research. BMJ. 2017;359:j5303. 10.1136/bmj.j5303. 29167093 10.1136/bmj.j5303

[42] Sonntag U, Brink A, Renneberg B, et al. GPs‘ attitudes, objectives and barriers in counselling for obesity – a qualitative study. Eur J Gen Pract. 2011;18(1):9–14. 10.3109/13814788.2011.627424. 22034942 10.3109/13814788.2011.627424

[43] Ogden J, Baig S, Earnshaw G, et al. What is health? GPs and patients worlds collide. Patient Educ Couns. 2001;45(4):265–9. 10.1016/s0738-3991(01)00128-8. 11755771 10.1016/s0738-3991(01)00128-8

[44] Ogden J, Flanagan Z. Beliefs of the causes and solutions of obesity: A comparison of gps and Lay people. Patient Educ Couns. 2008;71(1):72–8. 10.1016/j.pec.2007.11.022. 18201860 10.1016/j.pec.2007.11.022

[45] Klumbiene J, Petkeviciene J, Vaislavavicius V, et al. Advising overweight patients about diet and physical activity in primary health care: Lithuanian health behaviour monitoring study. BMC Public Health. 2006;6(30):30–6. 10.1186/1471-2458-6-30. 16478535 10.1186/1471-2458-6-30 PMC1382204

[46] Ärzte Zeitung. Krankenkassen fordern mehr strukturierte Behandlungsprogramme [Health insurance companies demand more structured treatment programs]. 2018. https://www.aerztezeitung.de/medizin/krankheiten/adipositas/article/955042/adipositas-krankenkassen-fordern-strukturierte-behandlungsprogramme.html [Cited 2024 Okt 10].

[47] Herbers J. Der Hausarzt Als ‚Abnehm-Coach‘ [The family Doctor as a ‘weight loss coach’]. 2013. https://www.allgemeinarzt-online.de/archiv/a/der-hausarzt-als-abnehm-coach-1571807 [Cited 2024 Okt 10].

[48] KBV [Kassenärztliche Bundesvereinigung]. DMP Adipositas. 2024. https://www.kbv.de/html/69609.php [Cited 2024 Okt 10].

[49] Wangler J, Jansky M. How can primary care be secured in the long term? – A qualitative explorative study from the perspective of general practitioners in Germany. Eur J Gen Pract. 2023;29(1):2223928. 10.1080/13814788.2023.2223928. 37387362 10.1080/13814788.2023.2223928 PMC10316722

[50] Greiner KA, Born W, Hall S, et al. Discussing weight with obese primary care patients: physicians and patient perceptions. J Gen Intern Med. 2008;23(5):581–7. 10.1007/s11606-008-0553-9. 18322760 10.1007/s11606-008-0553-9 PMC2324159

[51] Bortz J, Schuster C. Statistik für human- und sozialwissenschaftler [Statistics for human and social Scientists]. Heidelberg: Springer; 2010.

[52] Mayring P. Qualitative Inhaltsanalyse. Grundlagen und techniken [Qualitative content analysis: basics and Techniques]. Beltz: Weinheim; 2010.

[53] Wangler J, Jansky M. How are people with obesity managed in primary care? - results of a qualitative, exploratory study in Germany 2022. Arch Public Health. 2023;81(1):196. 10.1186/s13690-023-01214-z. 37957725 10.1186/s13690-023-01214-z PMC10641940

[54] Ryan L, Quigley F, Birney S, et al. Beyond the scale’: a qualitative exploration of the impact of weight stigma experienced by patients with obesity in general practice. Health Expect. 2024;27(3):e14098. 10.1111/hex.14098. 38859797 10.1111/hex.14098 PMC11165259

[55] Rurik I, Torzsa P, Ilyés I, et al. Primary care obesity management in Hungary: evaluation of the knowledge, practice and attitudes of family physicians. BMC Fam Pract. 2013;14(1):156. 10.1186/1471-2296-14-156. 24138355 10.1186/1471-2296-14-156 PMC3853764

[56] Turner LR, Harris MF, Mazza D. Obesity management in general practice: does current practice match guideline recommendations? Med J Aust. 2015;202(7):370–2. 10.5694/mja14.00998. 25877119 10.5694/mja14.00998

[57] Mazza D, McCarthy E, Carey M, et al. 90% of the time, it’s not just weight: general practitioner and practice staff perspectives regarding the barriers and enablers to obesity guideline implementation. Obes Res Clin Pract. 2019;13(4):398–403. 10.1016/j.orcp.2019.04.001. 31109793 10.1016/j.orcp.2019.04.001

[58] Trujillo-Garrido N, Bernal-Jiménez MA, Santi-Cano MJ. Evaluation of obesity management recorded in electronic clinical history: a cohort study. J Clin Med. 2020;9(8):2345. 10.3390/jcm9082345. 32717839 10.3390/jcm9082345 PMC7465947

[59] Abbott S, Parretti HM, Greenfield S. Experiences and perceptions of dietitians for obesity management: a general practice qualitative study. J Hum Nutr Diet. 2021;34(3):494–503. 33438804 10.1111/jhn.12855

[60] Durrer D, Pasi P, Peterli R, et al. Improving obesitymanagement: Insights from the ACTION Switzerland survey of people with obesity, physicians and dietitians. Clin Obes. 2025;15(2):e12716. 10.1111/cob.12716. 39511701 10.1111/cob.12716 PMC11907096

[61] Trujillo-Garrido N, Santi-Cano MJ. Motivation and limiting factors for adherence to weight loss interventions among patients with obesity in primary care. Nutrients. 2022;14(14):2928. 10.3390/nu14142928. 35889885 10.3390/nu14142928 PMC9316956

[62] ACTION. ACTION Study health care professional tool kit. 2018. https://www.rethinkobesity.com/content/dam/obesity/rethink-obesity/pdf-files/RESOURCES_ACTION_Study_HCP_Toolkit.pdf [Cited 2025 May 28].

[63] Sharma AM, Bélanger A, Carson V, et al. Perceptions of barriers to effective obesity management in canada: results from the ACTION study. Clin Obes. 2019;9(5):e12329. 10.1111/cob.12329. 31294535 10.1111/cob.12329 PMC6771494

[64] Tham KW, Ahmed A, Boonyavarakul A, et al. ACTION APAC: understanding perceptions, attitudes and behaviours in obesity and its management across South and Southeast Asia. Clin Obes. 2024;14(3):e12644. 10.1111/cob.12644. 38332544 10.1111/cob.12644

[65] Wadden TA, Volger S, Sarwer DB, et al. A two-year randomized trial of obesity treatment in primary care practice. N Engl J Med. 2011;365(21):1969–79. 10.1056/NEJMoa1109220. 22082239 10.1056/NEJMoa1109220 PMC3282598

[66] Osmundsen TC, Dahl U, Kulseng B. Enhancing knowledge and coordination in obesity treatment: a case study of an innovative educational program. BMC Health Serv Res. 2019;19(1):278. 10.1186/s12913-019-4119-9. 31046766 10.1186/s12913-019-4119-9 PMC6498688

[67] Verberne LDM, Nielen MMJ, Leemrijse CJ, et al. Recording of weight in electronic health records: an observational study in general practice. BMC Fam Pract. 2019;19(1):174. 10.1186/s12875-018-0863-x. 10.1186/s12875-018-0863-x PMC6240309 30447691

[68] Grima M, Dixon JB. Obesity – recommendations for management in general practice and beyond. Aust Fam Physician. 2013;42(8):532–41. 23971060

[69] Gunther S, Guo F, Sinfield P, et al. Barriers and enablers to managing obesity in general practice: a practical approach for use in implementation activities. Qual Prim Care. 2012;20(2):93–103. 22824562

[70] Forgione N, Deed G, Kilov G, Rigas G. Managing obesity in primary care: breaking down the barriers. Adv Ther. 2018;35(2):191–8. 10.1007/s12325-017-0656-y. 29349682 10.1007/s12325-017-0656-y PMC5818554

[71] Prüfer F, Joos S, Milksch A. Die Rolle des Hausarztes in der kommunalen Gesundheitsförderung – Eine qualitative studie [The role of general practitioners in community health promotion – A qualitative study]. Präv Gesundheitsf. 2015;10(2):180–5. 10.1007/s11553-015-0486-1.

[72] Ärzte Zeitung. DMP Adipositas – Änderung des Lebensstils im Fokus [DMP Obesity – Focus on Lifestyle Change]. 2024. https://www.aerztezeitung.de/Medizin/DMP-Adipositas-Aenderung-des-Lebensstils-im-Fokus-447906.html [Cited 2024 Okt 10].

[73] Malatzky C, Glenister K. Talking about overweight and obesity in rural Australian general practice. Health Soc Care Community. 2019;27(3):599–608. 10.1111/hsc.12672. 30311287 10.1111/hsc.12672

[74] Dahlhausen F, Zinner M, Bieske L, et al. Physicians’ attitudes toward prescribable mHealth apps and implications for adoption in germany: mixed methods study. JMIR Mhealth Uhealth. 2021;9(11):e33012. 10.2196/33012. 34817385 10.2196/33012 PMC8663495

[75] Buss VH, Barr M, Parker SM, et al. Mobile app intervention of a randomized controlled trial for patients with obesity and those who are overweight in general practice: user engagement analysis quantitative study. JMIR Mhealth Uhealth. 2024;12:e45942. 10.2196/45942. 38335014 10.2196/45942 PMC10891495

[76] Tan D, Zwar NA, Dennis SM, et al. Weight management in general practice: what do patients want? Med J Aust. 2006;185(2):73–5. 10.5694/j.1326-5377.2006.tb00474.x. 16842059 10.5694/j.1326-5377.2006.tb00474.x

[77] Ryan L, O’Donoghue G, Crotty M, Birney S, Heary C, Hanlon M, et al. Factors that influence general practitioners’ obesity-related clinical practices and determinants of behavior to target to promote best practice in obesity care: a qualitative exploration. Obes Sci Pract. 2024;10(5):e70012. 10.1002/osp4.70012. 39345781 10.1002/osp4.70012 PMC11438194

