# Supplementary material for: Prerequisites for primary care in obesity counselling and management: a quantitative, exploratory survey of general practitioners in the federal Republic of Germany
Source: BMC Prim Care. 2025 Oct 15;26:313. doi: 10.1186/s12875-025-03048-w (PMC12522577; doi:10.1186/s12875-025-03048-w)
Supplement: Supplementary file 1 — Supplementary Material 1. [file 12875_2025_3048_MOESM1_ESM.pdf]

Quantitative survey: Obesity Management in Primary Care

1. Try giving a rough estimate: Around what proportion of your patients have been affected by severe or very severe overweight or obesity over the last one or two years?

Around ___ percent

2. How far would you say the number of patients affected by severe or very severe overweight or obesity coming to your practice has increased over the last five to ten years? (Please enter the period between the time you began as a general practitioner and today if you've recently opened your general practice.) **Would you say the number has…**

◯ Increased a lot ◯ Increased somewhat ◯ Mild to moderate increase
◯ Not increased, remained the same ◯ Hard to say

3. In your opinion: How far does severe or very severe overweight or obesity present a challenge for the German healthcare system? Would you say it's a…

◯ Very great challenge ◯ Somewhat of a challenge ◯ Moderate or less of a challenge
◯ Minor challenge ◯ Hard to say

4. What are the most common causes or causal complexes for obesity in patients in your opinion or experience? A few points are enough.

____

____

____

5. What role do psychosocial problems play in obesity development or perpetuation in your opinion or experience?

◯ Major role ◯ Somewhat of a role ◯ Not much of a role ◯ No role ◯ Hard to say

6. How do you usually talk to patients about their excess weight? Do affected patients go to you specifically for their obesity and actively bring it up on their own, or do they come to your consultation for other reasons with the topic raised in a more incidental way or in connection with some other matter?

Affected patients come specifically because of obesity (such as to ask about therapy):

◯ Often ◯ Occasionally ◯ Rarely ◯ Never ◯ Hard to say

Other reasons for bringing up obesity incidentally:

◯ Often ◯ Occasionally ◯ Rarely ◯ Never ◯ Hard to say

7. When bringing up the topic of obesity for the first time with patients affected by obesity: What originally prompted the subject, what were the circumstances when you initially brought up obesity (such as a checkup, patient coming to see you to ask for advice on obesity)?

____

____

____

8. How far do you see general practitioners as the first point of contact for obesity management, treatment, or prevention (including lifestyle and weight advice)? Do you see it as the primary responsibility of a general practitioner to deal with obesity management in patients affected, or do you see that as the responsibility of specialists (such as diabetologists, nutritional consultants)? (This does not mean referring to specialists, but rather who you see as primarily responsible for disease management.)

◯ Mainly general practitioners responsible ◯ Mainly specialists responsible ◯ Hard to say

9. Depending on your answer, why are general practitioners especially suitable or less suitable as the first point of contact for patients? A few points are enough.

____

____

____

10. Considering the total number of patients affected by obesity that you see in regular general practice: How often do you provide lifestyle and weight counselling to patients affected by obesity?

◯ Often ◯ Occasionally ◯ Rarely ◯ Never (Please proceed to question 14)

11. And are these mostly one-off consultations or do they usually take place at different appointments, or continuously (patients coming back for follow-up appointments)?

◯ Usually one-off consultations ◯ Usually several appointments or continuously ◯ Hard to say

12. Which of the following points do you see as especially important in weight counselling? (You may choose multiple options)

◯ (Guideline-compliant) diagnosis ◯ Possible obesity causes ◯ Discussing the patient's personal life situation ◯ Explicitly bringing up the consequences of being overweight ◯ General information on the principles of losing weight (gradual vs. acute weight loss) ◯ Handing out information materials, quoting sources ◯ Specific suggestions and recommendations for empowering patients to lose weight on their own ◯ Specific advice on exercise ◯ Specific advice on diet ◯ Psychosocial situation of the patient, stabilisation or improvement ◯ Agreement on individual (weight loss) targets with monitoring at follow-up appointments ◯ Referral to specialist care/treatment services (specialists, nutritionists, specialised centre if necessary)

Other, please elaborate:

____

13. Considering the total number of patients affected by obesity that you have provided lifestyle and weight counselling for: How often have you…

Recommended specific services (such as dietary counselling, health insurance services, sports courses) to patients affected by obesity:

◯ Often ◯ Occasionally ◯ Rarely ◯ Never ◯ Hard to say

If so, please specify:

____

I've referred patients with obesity to specific health services (such as out of collaboration with these providers and services):

◯ Often ◯ Occasionally ◯ Rarely ◯ Never ◯ Hard to say

If so, please specify:

____

Referred patients with obesity to a specialist or specific healthcare facility for further lifestyle or weight counselling or obesity management:

◯ Often ◯ Occasionally ◯ Rarely ◯ Never ◯ Hard to say

Recommended certified digital health apps (DiGA apps) and/or other health apps for support or prevention

◯ Often ◯ Occasionally ◯ Rarely ◯ Never ◯ Hard to say

14. How do patients affected by obesity react when you first ask them about their overweight in your experience? What have your main experiences been in this situation?

____

____

____

15. And how do patients affected by obesity react to you providing lifestyle and weight counselling when you recommend weight reduction/lifestyle changes (behaviour, motivation, willingness)?

____

____

____

16. Considering the total number of patients affected by obesity that you see in regular general practice: How often do you take on responsibility in obesity management, that is, how often do you involve yourself in therapeutic measures in the medium to long term for your patients?

◯ Often ◯ Occasionally ◯ Rarely ◯ Never (Please proceed to question 31)

17. **Do you usually take the lead or primary responsibility for therapeutic measures, that is, do you play a main role in disease management, or do you leave this to specialists and follow their instructions?** (This is about whether you play the central role in disease management and make appropriate decisions, not whether or not other specialists are involved as well.)

◯ Main responsibility ◯ Follow specialist instructions ◯ Hard to say, varies widely

18. **How easy or difficult is it for your patients to accept obesity management or therapy in your experience?** (This refers to general willingness to engage in therapy.) **In your opinion:**

◯ Very difficult ◯ Somewhat difficult ◯ Somewhat easy ◯ Very easy ◯ Hard to say

19. **What would you say is the main reason in your experience?**

____

____

20. **How easy or difficult is it for you to keep your patients with obesity motivated in disease management towards achieving lifestyle changes or noticeable weight loss?**

◯ Very difficult ◯ Somewhat difficult ◯ Somewhat easy ◯ Very easy ◯ Hard to say

21. **What would you say is the main reason in your experience?**

____

____

22. **How would you generally assess your patients' adherence to counselling and therapy?** (This refers to adherence to and compliance with an agreed therapy that has already begun.) **In your experience, is adherence…**

◯ Very high ◯ Somewhat high ◯ Somewhat low ◯ Very low ◯ Hard to say

23. **Where do you place your focus in planning therapy towards helping obese patients lose weight? Please rate the importance of the following elements in your opinion.**

Change in diet, diet therapy

◯ Very high ◯ Somewhat high ◯ Somewhat low or not at all ◯ Hard to say

Exercise therapy, promoting physical exercise, sports

◯ Very high ◯ Somewhat high ◯ Somewhat low or not at all ◯ Hard to say

Behavioural therapy (improving behavioural patterns and changing them in the long term)

◯ Very high ◯ Somewhat high ◯ Somewhat low or not at all ◯ Hard to say

Adjuvant drug therapy (such as orlistat, liraglutide)

◯ Very high ◯ Somewhat high ◯ Somewhat low or not at all ◯ Hard to say

Psychosocial support

◯ Very high ◯ Somewhat high ◯ Somewhat low or not at all ◯ Hard to say

Organising information and materials for self-help

◯ Very high ◯ Somewhat high ◯ Somewhat low or not at all ◯ Hard to say

Other, please elaborate:

____

24. When you're drawing up an actual therapy plan: Do you usually work with intermediate targets in addition to setting a final target with your patient?

◯ Yes, usually several intermediate targets ◯ Yes, usually one intermediate target ◯ No, usually not

25. What challenges have you faced in implementing structured and effective obesity management plans?

____

____

____

26. Do you have a specific approach or strategy towards motivating patients during disease management to maximise therapy outcomes? What do you see as especially important in this regard?

____

____

____

27. Are surgical measures an option that you would include in obesity management under your responsibility and recommend to your patients?

◯ Yes, without restrictions ◯ Yes, with restrictions ◯ Only in extreme cases ◯ No

28. Please state any reasons why or why not.

____

____

____

29. **Taking stock of the situation: How would you assess the medium and long-term effects you've observed obesity management for your patients based on your experience so far?** (This means how well you think the therapies worked, or how satisfied you've been with them.) **How have the outcomes been in terms of achieving weight and lifestyle changes?**

◯ Very favourable ◯ Somewhat favourable ◯ Rather poor ◯ Very poor ◯ Hard to say

30. **What do you see as the main reasons for this?**

____

____

____

31. **Physicians have been allowed to prescribe certified digital health apps (DiGA apps) to patients since 2020. The aim of these prescription-only digital health apps is to contribute towards improving disease diagnosis and treatment as well as prevention. These apps are considered as medical products, unlike ordinary health apps that are freely available to patients without prescription. The German Federal Institute for Drugs and Medical Devices (BfArM) audits potential digital health apps and, if they pass the audit, adds them to the *DiGA-Verzeichnis*, a directory of health apps certified and covered by the German national health system.**

Have you already prescribed DiGA apps for patients with obesity, or do you plan to in the near future? Can you generally imagine doing so, or is prescribing DiGA apps out of the question for you?

◯ Already prescribed ◯ I plan to => Please go directly to question 36

◯ I can imagine it => Please go directly to question 36

◯ Out of the question => Please go directly to question 36

32. **Considering the total number of patients with obesity whose management you've responsibility for in the last one to two years: How often have you prescribed certified digital health apps (DiGA apps) to patients as part of their therapy?**

◯ Often ◯ Occasionally ◯ Rarely ◯ Hard to say, don't know

33. **Casting your mind back, which DiGA apps have you mostly prescribed to patients?**

◯ Oviva Direkt for obesity ◯ Zanadio

◯ Other:____

34. **Which favourable effects on the health status have you already seen as a result of DiGA apps in your patients with obesity?** (You may choose multiple options)

◯ Improved health awareness and education

◯ Increased compliance, such as taking their medications

◯ Improvement in self-management (such as in chronic disease)

◯ Improvement in self-management, such as in chronic disease

◯ Increase mobility and joy of exercise

◯ Substantial weight reduction (such as BMI, abdominal circumference, waist circumference)

◯ Stable decrease in blood sugar (HbA1c)

◯ Regression of metabolic syndrome

◯ Decrease in psychological issues and sequelae (such as depression)

◯ Prevention of sequelae (such as diabetic foot syndrome, CHD)

◯ Decrease in complications (such as hypoglycaemia)

◯ Elimination of need for more severe management options (such as insulin therapy)

◯ Other: ____

35. **How would you assess the overall benefit of DiGA apps in disease management and obesity treatment based on your experience so far?**

◯ Very useful ◯ Somewhat useful ◯ Mildly useful ◯ Not at all useful ◯ Hard to say

36. **Various guidelines and additional guides and clinical recommendations are available for obesity. Which of the following guidelines are you familiar with, which ones have you already applied?** (If not known: leave this line blank.)

	Known, frequently used	Known, occasionally used	Known, rarely used	Known, not yet used
Interdisciplinary obesity S3 guideline on prevention and therapy	◯	◯	◯	◯
DEGAM clinical recommendation: Primary care for patients with obesity/overweight	◯	◯	◯	◯
Obesity prevention and therapy for children and young people	◯	◯	◯	◯

S3 guideline: Surgery for obesity (and metabolic diseases)	◯	◯	◯	◯
Guideline on obesity and pregnancy	◯	◯	◯	◯
Guideline on non-alcoholic fatty liver disease	◯	◯	◯	◯
Guideline on type 1 diabetes therapy [Nicht type 2?]	◯	◯	◯	◯
Nutritional recommendations diabetes mellitus treatment – recommendations for protein intake	◯	◯	◯	◯
Patient guideline on obesity diagnosis and treatment	◯	◯	◯	◯
Patient guideline on surgery for obesity (and metabolic diseases)	◯	◯	◯	◯

37. Overall, how useful or helpful would you rate the obesity guidelines you have used so far?

◯ Very useful ◯ Somewhat useful ◯ Mildly useful ◯ Not at all useful
◯ Hard to say or do not used any obesity guidelines so far

38. And where do you see deficits and problems in existing guidelines?

____

____

____

39. Do you have practice staff members with special training in topics such as obesity, weight reduction, nutrition, exercise, or similar? (This refers to taking the appropriate training courses.)

◯ Yes, most have training ◯ Yes, some have training ◯ Yes, one has training ◯ No

40. What measures do you think would make an effective contribution to improving obesity treatment and management and making it even more effective for patients with obesity in general practice? What needs to change or improve?

____

____

____

41. **A decision was recently made to establish an obesity disease management programme. How far do you see this as an effective contribution to improving outpatient care for obesity or obesity management?**

◯ Very effective ◯ Somewhat effective ◯ Mildly effective ◯ Not effective at all ◯ Hard to say

42. **Please state any reasons why or why not.**

____

____

____

43. **How interested are you in participating in the obesity management programme (DMP Adipositas)?**

◯ Very much ◯ Somewhat ◯ Not so much ◯ No interest at all

We would like to ask you for some information for statistical purposes. As with the rest of the questionnaire, the information you give will of course be treated in strict confidence and anonymity.

Are you…

◯ Male ◯ Female ◯ Diverse

Your **age**: ________

Where is your medical practice located? In a municipality or city with a population of…

◯ more than 100,000 ◯ 20,000 to 100,000 ◯ 5,000 to 20,000 ◯ less than 5,000 inhabitants

Which of the following most accurately describes your **medical practice**?

◯ Individual practice (you are the only doctor) ◯ Individual practice with employed doctors on staff

◯ Group practice ◯ Medical care centre or polyclinic ◯ Other

How many patients come to your practice for treatment each quarter?

◯ 500 to 750 ◯ 751 to 1,000 ◯ 1,001 to 1,500 ◯ 1,501 to 2,000 ◯ More than 2,000

Which of the following **additional qualifications** do you have?

◯ Diabetology ◯ Nutritional medicine ◯ Physical therapy ◯ Psychotherapy

◯ Social medicine ◯ Sports medicine

Thanks for your time!

Is there anything else you would like to tell us? Use this space for suggestions, comments, and criticisms.

____

____

____
